# Chromosomal localization and molecular marker development of the lipopolysaccharide and beta-1,3-glucan binding protein gene in the Zhikong scallop *Chlamys farreri* (Jones et Preston) (Pectinoida, Pectinidae)

**DOI:** 10.1590/S1415-47572010005000015

**Published:** 2010-03-01

**Authors:** Pin Huan, Xiaojun Zhang, Fuhua Li, Yang Zhang, Cui Zhao, Jianhai Xiang

**Affiliations:** 1 Key Laboratory of Experimental Marine Biology, Institute of Oceanology, Chinese Academy of Sciences, QingdaoP.R. China; 2 Graduate University of Chinese Academy of Sciences, BeijingP.R. China; 3 Department of Soil Crop Sciences, Texas AM University, College Station, TXUSA

**Keywords:** *Chlamys farreri*, LGBP, *fluorescence in situ hybridization (FISH)*, indel, SNP

## Abstract

Zhikong scallop *Chlamys farreri* (Jones et Preston) is an economically important species in China. Understanding its immune system would be of great help in controlling diseases. In the present study, an important immunity-related gene, the Lipopolysaccharide and Beta-1,3-glucan Binding Protein (LGBP) gene, was located on *C. farreri* chromosomes by mapping several *lgbp*-containing BAC clones through fluorescence *in situ* hybridization (FISH). Through the localization of various BAC clones, it was shown that only one locus of this gene existed in the genome of *C. farreri*, and that this was located on the long arm of a pair of homologous chromosomes. Molecular markers, consisting of eight single nucleotide polymorphism (SNPs) markers and one insertion-deletion (indel), were developed from the LGBP gene. Indel marker testing in an F1 family revealed slightly distorted segregation (p = 0.0472). These markers can be used to map the LGBP gene to the linkage map and assign the linkage group to the corresponding chromosome. Segregation distortion of the indel marker indicated genes with deleterious alleles might exist in the surrounding region of the LGBP gene.

## Introduction

The first step in the immune response for organisms is to recognize invading molecules. This is generally the function of pathogen recognition proteins (PRPs), capable of recognizing the specific molecules existing in the invading organism through pathogen-associated molecular patterns (PAMPs) (Janeway Jr, 1989). After recognizing specific PAMPs, including lipopolysaccharide (LPS), peptidoglycan (PG) and beta-1,3-glucans (BG), PRPs initiate downstream immune responses. These proteins are essential for the invertebrates, as they lack adaptive immune system. The lipopolysaccharide and beta-1,3-glucan binding protein (LGBP), as a form of PRP, can recognize both LPS existing on the surface of Gram-negative bacteria, as well as BG on the surface of fungi. Furthermore, it can trigger off activation of the prophenoloxidase (proPO) system, thereby leading to the synthesis of antimicrobial proteins and peptides, after binding with LPS or BG (Lee, 2000; Sritunyalucksana and Söderhäll, 2000). The full length cDNA of LGBP gene has been identified in many invertebrates, including *Bombyx mori* (Lepidoptera, Bombycidae) (Lee *et al.*, 1996), *Drosophila melanogaster* (Diptera, Drosophilidae) (Kim *et al.*, 2000), *Pacifastacus leniusculus* (Decapoda, Astacidae) (Lee *et al.*, 2000), *Fenneropenaeus chinensis* (Decapoda, Penaeidae) (Liu *et al.*, 2007) and *Litopenaeus vannamei* (Decapoda, Penaeidae) (Cheng *et al.*, 2005), Research has revealed pronounced LGBP affinity to LPS and BG.

The Zhikong scallop *Chlamys farreri* (Jones et Preston) (Pectinoida, Pectinidae) is an economically important species in China. Much research has been dedicated to this species and appreciable progress made (Li *et al.*, 2005; Wang *et al.*, 2005a; Zhan *et al.*, 2007, 2008; Zhang *et al.*, 2007a, 2008). Nevertheless, over the last decade, problems with disease have assumed increasing severity, attaining high mortality. An understanding of the immune system would be extremely beneficial in dealing with this problem. To date, many immune-related genes have been cloned and their expressions analyzed (Su *et al.*, 2004; Gao *et al.*, 2007; Qiu *et al.*, 2007a, b; Wang *et al.*, 2007; Yu *et al.*, 2007; Zhang *et al.*, 2007a, 2008). LGBP gene expression in *C. farreri* was significantly up-regulated following *Vibrio anguillarum* challenge (Vibrionales, Vibrionaceae), this indicating a possibly important role during infection with gram-negative bacteria (Su *et al.*, 2004).

Nevertheless, the above-mentioned studies on immune related genes in *C. farreri* have mainly focused on gene expression at transcription or translation levels. Results at the genomic DNA level have seldom been reported, although they could provide information on gene regulation and localization, which is especially important for genetic breeding. The lack of several important resources, such as cell lines and large-insert genomic DNA libraries, limits research thereof. Recently, the situation improved with the construction of a species specific fosmid library (Zhang *et al.*, 2007b) and two BAC libraries (Zhang *et al.*, 2008) on *C. farreri*.

Gene localization on genetic, physical and cytogenetic maps could provide basement support for fundamental and applied research. In our previous work, *lgbp*-containing BAC clones were screened out from the BAC library of *C. farreri* through over-go screening (Zhang *et al.*, 2008). In the present work, BAC clones containing LGBP genes were successfully mapped to *C. farreri* chromosome by both single-color and double-color fluorescence *in situ* hybridization (FISH), whereby various molecular markers in LGBP gene were developed for genetic mapping. This could be an aid in expanding knowledge on the innate immune system and in promoting the integration of physical, genetic and cytogenetic maps of *C. farreri* in the near future.

## Material and Methods

###  Chromosome preparation

Chromosomes were prepared from larvae of *C. farreri*. Larvae were treated with 0.01% colchicine for 2 h, and then transferred into 0.075 mol/L of KCl for a further 15 min. After hypotonicity, the larvae were fixed twice in Carnoy solution (methanol: acetic acid, 3: 1), for 15 min each time, and then stored in storage solution (methanol: acetic acid, 1: 1) at -20 °C. Just before use, the fixed larvae were dissolved in freshly prepared 50% acetic acid, dropped onto clean slides, and then air dried.

###  Validation of the BAC clones

Six BAC clones, *viz*., CFB094J04, CFB066B03, CFB040L24, CFB183I08, CFM008L23 and CFM005H15, were identified as *lgbp*-containing clones in our previous study, by using over-go screening (Zhang *et al.*, 2008). Identity was further confirmed through PCR reactions. A couple of primers were designed based on the LGBP gene cDNA sequence of *C. farreri* (GenBank No. AY259542). The forward primer (CFLGBPF1) was 5'- GGGAACGCATACATCAT-3', and the reverse (CFLGBPR2) was 5'- CGATCCGTGGTAAGTGT-3'. PCR reactions were carried out using both BAC plasmids and genomic DNA as templates. The PCR products were cloned into host bacteria (*E. coli*., Top10) and sequenced by means of an ABI 3730 sequencer. The obtained sequences were analyzed by BLASTN search against the NCBI non-redundant (nr) database.

###  Plasmid extraction and probe synthesis

For plasmid extraction, the *lgbp*-containing BAC clones were cultured overnight at 250 rpm and 37 °C. The bacteria were then collected by centrifugation at 3000 g for 15 min. BAC plasmids were purified with a NUCLEOBOND AX 100 kit (Machery-Nagel), examined by agarose gel electrophoreses, and after quantified with a spectrophotometer.

One microgram of BAC plasmid was used for probe synthesis. Digoxigenin or biotin-labeled probes were generated using the corresponding nick translation labeling kit from the Roche Company. *Cot-1* DNA, which was used as competitive DNA in FISH, was prepared according to the method described by Zwick *et al.* (1997).

###  Fluorescence in situ hybridization

For hybridization, 1 μg of probe was co-precipitated with 10 μg of *Cot-1* DNA and 50 μg of salmon sperm DNA, and then dissolved in 60 μL of blank hybridization buffer (50% formamide, 2x SSC, 10% dextran sulfate, 50 mmol/L phosphate buffer, pH 7.0), this yielding a hybridization buffer with a probe concentration of 16.67 ng/μL. For double-color FISH, digoxigenin and biotin-labeled probes were mixed equally and then co-precipitated. These were dissolved as described above.

Chromosome slides were pre-treated using 1.6% pepsin at 37 °C for 30 min and washed in 2x SSC for at least 2 min. They were then denatured in 70% formamide at 68 °C for 2 min. To suppress the repetitive sequences that might exist in the probes, the hybridization buffer was pre-annealed at 37 °C for 1 h after denaturation at 78 °C for 3 min. Ten microliters of pre-annealed hybridization buffer was added to each slide, whereupon a coverslip was applied. The slide was sealed and incubated at 37 °C for 14 to 22 h in a humid box. Immunodetection was carried out using mouse-anti-digoxigenin and FITC-rabbit-anti-mouse and FITC-goat-anti-rabbit (for digoxigenin-labeled probes), or rhodamine-avidin and biotin-anti-avidin (for biotin-labeled probes), respectively. For double-color FISH, the antibodies of each layer were mixed and then incubated together. DAPI (4',6-diamidino-2-phenylindole) was used to counter-stain the chromosomes. Slides were observed under a Nikon 80i microscope equipped with a cool CCD. Pictures were merged and edited using NIS-element (Nikon) and Photoshop (Adobe) software.

###  Molecular marker development

To develop SNP markers, CFLGBPF1 and CFLGBPR2 primers were used for amplifying fragments from *C. farreri* genomic DNA. PCR products were directly sequenced using an ABI 3730 sequencer. SNPs were identified through the presence of two sequence peaks at each locus in the sequencing trace files. To eliminate potentially false positives, the DNA from two individuals which had been proved to be highly heterozygous in a previous study, and a DNA sample prepared by mixing the DNA of 30 randomly selected individuals, were used as PCR templates. Each SNP marker was nominated as the base of one allele followed by its locus and the base of the other allele. For example, G169A refers to a G/A SNP marker on locus 169 of the reference sequence.

In this study, comparative analysis revealed many insertion-deletion variations of sequences amplified by CFLGBPF1 and CFLGBPR2 primers. Based on these variations, another reverse primer, CFLGBPR4 (5'- TCCAGGTCCAGCGTGTCG-3'), was used to generate an indel marker combining with CFLGBPF1. The location of CFLGBPR4 was carefully designed to ensure that, in the expected PCR products amplified from different alleles, variations in length could be about 50 bp. DNA samples of 13 randomly selected individuals of *C. farreri* were used as templates to guarantee that different alleles of the indel marker were distinguishable, one from the other, on 1.5% agarose gel electrophoresis.

The indel marker segregation pattern was explored through genotyping one F1 family with 105 progenies. PCR products of homogenous individuals with only one amplified fragment were marked ‘AA' (only the shorter fragment), or ‘aa' (only the longer fragment), and those of heterogeneous individuals, with both fragments amplified, were marked “Aa”. The number of individuals with each genotype was calculated, and the chi-square test was applied to detect whether the heredity of the indel marker coincided with the Mendel heredity principle.

## Results

###  Validation of BAC clones by PCR

All the six *lgbp*-containing BAC clones screened by over-go hybridization, *viz*., CFB094J04, CFB066B03, CFB040L24, CFB183I08, CFM008L23 and CFM005H15, were confirmed by PCR reactions using the primers CFLGBPF1 and CFLGBPR2. A BLASTN search of obtained sequences confronted with the NCBI non-redundant (nr) database showed that all the six clones bore the LGBP gene. The obtained sequences were submitted to NCBI, with accession numbers FJ434687- FJ434690.

###  Fluorescence *in situ* hybridization

**Figure 1:**
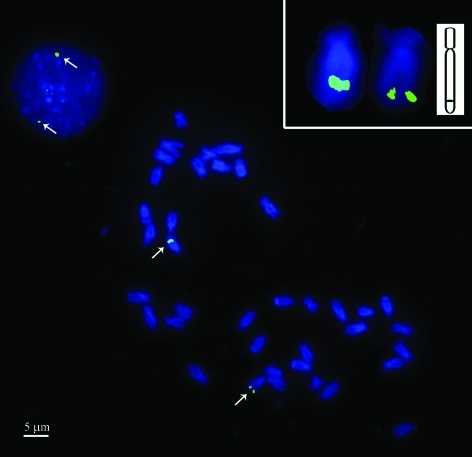
The LGBP gene was mapped to the distal end of the long arm of one pair of chromosomes and also the interphase nucleus of *Chlamys farreri* (Jones et Preston). Signals are indicated by arrows. The inserted picture at the top right-hand corner shows detailed figures of *lgbp*-bearing chromosomes and the sketch map of the hybridization pattern of the signal and chromosome. Bar = 5 μm.

One *lgbp*-containing BAC clone (CFB094J04) was first used for FISH. This clone, successfully mapped on the interphase nucleus and chromosome of *C. farreri* (Figure 1), was localized at the distal ends of the long arms of a pair of homologous chromosomes, of a total of 38. Based on measurements, the *lgbp*-bearing chromosomes were characterized with an arm-length ratio of 2.8.

**Figure 2:**
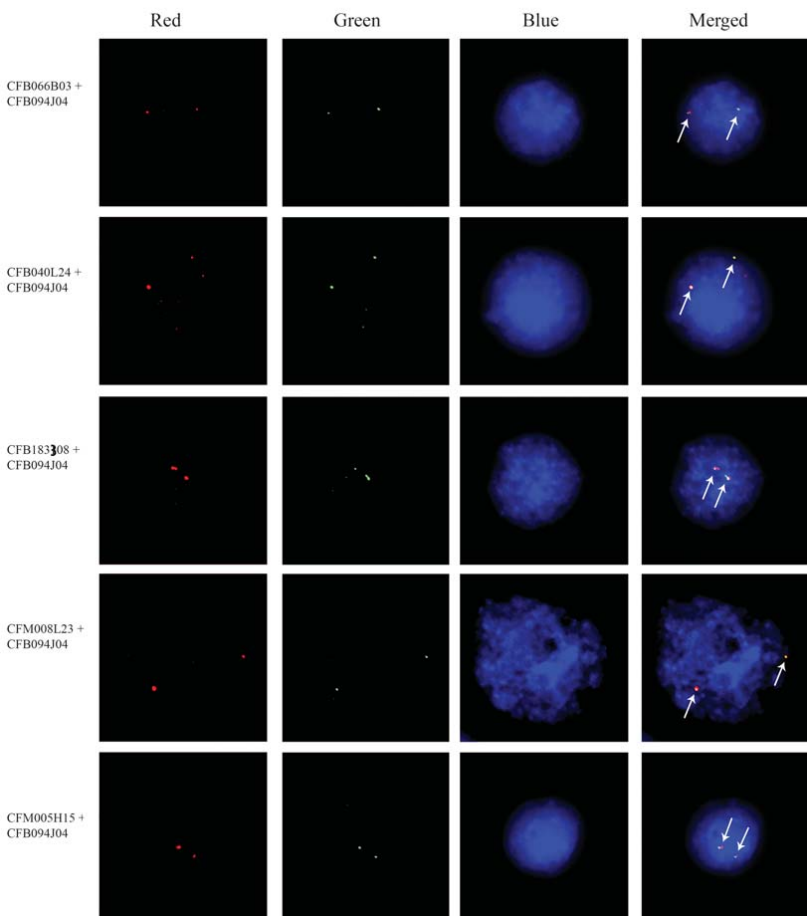
Double-color FISH showed the six *lgbp*-containing BAC clones co-localized at the same site on the *c. farreri* genome. The red, green and blue channels in the pictures were recorded separately and merged to obtain the final figures. The green signals indicate the localization of clone CFB094J04, which was mapped first using single color FISH, whereas the red signals of each set indicate that of the other five clones, respectively. The signals are indicated by arrows in the merged figures. Bar = 5 μm.

In order to confirm whether all the screened BAC clones were located on the same pair of chromosomes, CFB094J04 was co-hybridized with each of the other five *lgbp*-containing clones, CFB066B03, CFB040L24, CFB183I08, CFM008L23 and CFM005H15, through double-color FISH. Co-localization of CFB094J04 with each of the five was confirmed by the probes being capable of generating merged signals in each case (Figure 2). Thus, it was concluded that all the six *lgbp*-containing clones were located at the same site in the genome.

###  Sequence analysis and marker development

**Figure 3:**
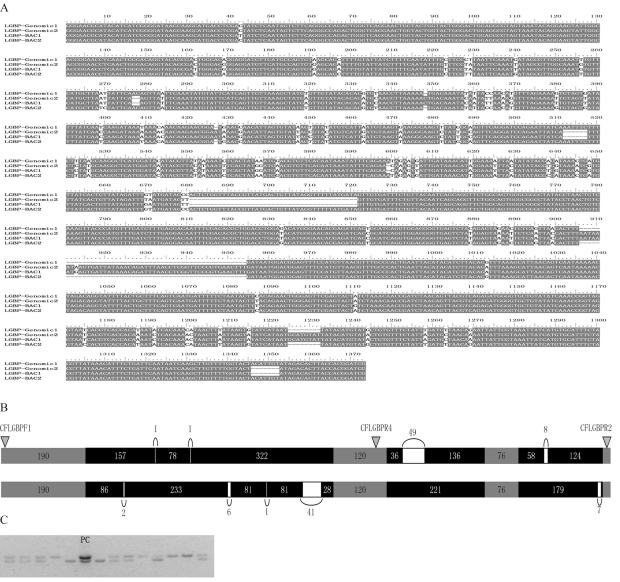
Sequence variations among partial sequences of *lgbp*, and the development of an indel marker. a. The multi-alignment of sequences amplified from BAC plasmids (LGBP-BAC1 and LGBP-BAC2) and genomic DNA (LGBP-genomic1 and LGBP-genomic2), showing insertion-deletion variation and several nucleotide mutations. b. Organization of the two variants; the numbers stand for fragment length (bp). The location of three primers (CFLGBPF1, CFLGBPR2 and CFLGBPR4) is indicated by triangles. c. Indel marker testing by using 13 randomly selected DNA samples showed the PCR products of different variants successfully separated from each other. A mixture of the two variants was used as positive controls (Lane PC).

Four sequences amplified from the CFLGBPF1 and CFLGBPR2 primers were obtained from both BAC clones and genomic DNA (FJ434687-FJ434690). Through analyzing these sequences, as well as the corresponding cDNA sequence of LGBP genes, it was concluded that the four consisted of four exons and three introns. Through further multi-alignment analysis, they were classified into two types: type 1 with a length of 1314 bp and type 2 with 1316 bp (Figures 3a and 3b). The varition in length between type 1 and type 2 were caused by nine insertion/deletions in the introns, with lengths ranging from 1 bp to 49 bp (Figure 3b). There were four deletions in type 1 and five in type 2.

**Figure 4:**
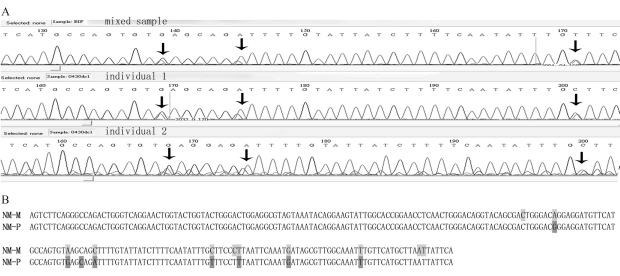
SNPs were discovered in a 224-bp long fragment by directly sequencing PCR products from both mixed DNA samples and DNA samples of single individuals. a. Sequencing trace files showed two sequence peaks at each SNP site. SNPs were coincident among the three samples as indicated by arrows. b. Comparative analysis of the nucleotide mutations revealed by multi-alignment (NM-M, marked in light gray) and the SNPs discovered by sequencing PCR products (NM-P, marked in dark gray).

Up to 77 nucleotide mutations were revealed through multi-alignment analysis (Figure 3a). To develop SNP markers, PCR products amplified from *C. farreri* genomic DNA were directly sequenced. Results are shown in Figure 4. Apart from the continuous signal confusions caused by the insertion-deletion variations, 224-bp sequences were readable in the sequencing-trace files. Eight SNPs were revealed in this region through the detection of two peaks at one and the same location, whereas there was only a single sequence peak at other locations (Figure 4a). Taking the sequence with the GenBank accession number FJ434689 as reference, they were denominated G169A, G191A, C194G, A197C, T223C, A229T, T240G and G256T. Among these, one (G169A) was located in the exons (total 138 bp) and seven in the introns (total 86 bp). Sequence translation revealed that the mutation at locus 169 in the coding region did not generate a mutation in amino acids (AGG → AGA, Arg).

Through comparative analysis, it was evident that seven of the SNPS detected in PCR product sequencing were coincident with the nucleotide mutations revealed by multi-alignment (Figure 3a). One, *viz.*, C194G, was only detected in PCR product sequencing, whereas four nucleotide mutations were only revealed through multi-alignment and not by sequencing (Figure 4b).

One indel marker was obtained through amplification by CFLGBPF1 and CFLGBPR4 primers. The reverse primer CFLGBPR4 was located in exon 2, thus PCR products of the two types would be 782 bp and 830 bp, respectively. Indel marker testing, using 13 randomly selected individuals, showed that the PCR products of the two genotypes could be separated one from the other in 1.5% agarose gel electrophoresis (Figure 3c).

A family of *C. farreri*, consisting of both parents and 105 progenies, was employed to provide evidence of the indel marker segregation pattern during heredity. The data showed that the number of progeny with genotype AA, aa and Aa was 26, 16 and 63 respectively, while both parents were heterozygous (Aa). Chi-square testing revealed the occurrence of slight segregation distortion (p = 0.0472, p < 0.05). Homozygous deficiency was detected, since the number of individuals with genotype aa (16) was significantly less than the expected (26.25).

## Discussion

In the present work, we localized *lgbp*-containing BAC clones on *C. farreri* chromosomes through FISH. When localizing specifically CFB094J04 in either the chromosome metaphase or the nucleus interphase, the result was the same. Co-localization of the six *lgbp*-containing BAC clones showed that LGBP genes are located on one chromosome in *C. farreri*. To our knowledge, this is the first report on mapping immune related genes on these chromosomes in particular.

Functionally related genes might be physically linked, and could thus form gene clusters. For instance, in human beings three transglutaminases form a gene cluster on chromosome 15q15 (Grenard *et al.*, 2001), whereas in *Drosophila*, six homeobox genes, involved in mesodermal patterning and differentiation programs, form a gene cluster at 93DE on the third chromosome (Jagla *et al.*, 2001). The immune system of *C. farreri* involves a vast number of functionally related genes (Su *et al.*, 2004; Gao *et al.*, 2007.; Qiu *et al.*, 2007a, b; Wang *et al.*, 2007; Yu *et al.*, 2007; Zhang *et al.*, 2007a, 2008). As things stand, it is quite reasonable to assume the existence of a certain number of gene clusters. So far, no physical linkage of these genes has been reported. FISH mapping of gene-containing BAC clones provides an important approach in exploring whether there are gene clusters among immune related genes. Based on large-insert libraries and double-color FISH, as presented in this study, many other immune related genes could be screened and mapped in the near future. The discovery of physical linkages among these genes would enlighten further research.

The localization of BACs facilitates chromosome identification in *C. farreri*, especially through FISH, which itself is both efficient and transferable by being sequence-specific. The construction of BAC libraries in *C. farreri* opens up vast possibilities for this identification process, by the abundance of resultant probes. Previous reports have already shown that FISH techniques together with large-insert libraries could be efficiently used, hereto. In *Crassostrea virginica* (Ostreoida, Ostreidae), ten chromosomes were identified through the localization of nine bacteriophage P1 clones together with the configuration characters of the chromosomes (Wang *et al.*, 2005b), and in *C. farreri*, eight of 19 chromosomeswere identified through the localization of eight fosmid clones (Zhang *et al.*, 2007c). Furthermore, the *lgbp*-bearing chromosome was successfully identified through locating *lgbp*-containing BACs with FISH. In the latter case, a larger number would have been if more BACs had been localized. Furthermore, FISH techniques are extremely helpful in the construction of integration maps through using molecular markers as bridges. In potatoes, all the linkage groups were assigned to the corresponding chromosomes by mapping 12 BAC clones which contained RFLP markers genetically mapped to the chromosomes (Dong *et al.*, 2000). Thus, it is possible to develop molecular markers in BAC clones of *C. farreri* for further application in constructing integration maps.

Molecular markers, including AFLP and SSR markers, have been developed in *C. farreri*, in the course of many studies (Li *et al.*, 2005; Wang *et al.*, 2005a; Zhan *et al.*, 2007; Zhan *et al.*, 2008**).** Herein, we developed several markers applicable to linkage mapping in *C. farreri* LGBP gene. On combining this with information from FISH mapping, LGBP gene mapping would assign the linkage group to the corresponding chromosome.

SNPs frequency and their distribution pattern in LGBP genes of *C. farreri* are both impressive. Altogether, we obtained eight SNP markers in a 224 bp-long region, an average length of 28 bp each. Although only a short fragment was analyzed in this study, it still reflected the high abundance of SNPs in the *C. farreri* genome, thus possibly implying the likelihood of developing these markers in any given region therein. Moreover, it was also observed that more markers are located in introns than in exons. Among the nine markers in this study, only one was located in a coding region (totally 138 bp), whereas the other seven, as well as the indel marker itself, were located in the non-coding region (a total of 86 bp), thereby reflecting the selective pressures which might be playing an important part in the evolution of the organism. The distribution model of molecular markers in coding and non-coding regions is important for marker development in the construction of genetic maps and their integration. The putative roles of molecular markers are important in selective breeding. Among the nine SNP markers identified in the present study, only one (G169A) was detected in the coding region, and proved to be a silent mutation. The role of G169A may be illustrated in future research.

Indel marker segregation was revealed to be slightly distorted through genotyping an F1 family of *C. farreri*. Segregation distortion in this scallop had already been revealed in previous studies (Li *et al.*, 2005; Wang *et al.*, 2005a). Artificial errors could account for this. In the present study, the possibility of false positives could not be excluded, since only a relatively small population (105 progeny) was used and the p value was close to 0.05 (p = 0.0472). On the other hand, and pending future confirmation, homozygote deficiency in indel marker heredity could be instructive in further research. In oysters, homozygote deficiency was explained by the high genetic load and selection against deleterious recessive mutations (Launey and Hedgecock, 2001). Accordingly, various alleles were hypothesized to be deleterious when they were homogenous (Hubert and Hedgecock, 2004). It is reasonable to assume the same might occur in *C. farreri*, due to the relatively close evolutional relationship. Therefore, it is instructive to observe a significant reduction in the F1 family “aa” genotype, since this may indicate genes with deleterious recessive alleles linked with the indel marker. In a previous study, LGBP has been shown to play crucial roles in infections through up-regulated expression immediately after *Vibrio anguillarum* challenge (Su *et al.*, 2004). Therefore, segregation distortion of the indel marker in LGBP genes provides functional illustration, although proof remains concealed.
